# Generation and Characterization of an Immortalized Human Esophageal Myofibroblast Line

**DOI:** 10.1371/journal.pone.0153185

**Published:** 2016-04-07

**Authors:** Chao Niu, Uday Chauhan, Matthew Gargus, Anisa Shaker

**Affiliations:** Department of Medicine, Division of Gastroenterology and Hepatology, Keck School of Medicine of USC, Los Angeles, California, United States of America; Shiga University of Medical science, JAPAN

## Abstract

Stromal cells with a myofibroblast phenotype present in the normal human esophagus are increased in individuals with gastro-esophageal reflux disease (GERD). We have previously demonstrated that myofibroblasts stimulated with acid and TLR4 agonists increase IL-6 and IL-8 secretion using primary cultures of myofibroblasts established from normal human esophagus. While primary cultures have the advantage of reflecting the in vivo environment, a short life span and unavoidable heterogeneity limits the usefulness of this model in larger scale in vitro cellular signaling studies. The major aim of this paper therefore was to generate a human esophageal myofibroblast line with an extended lifespan. In the work presented here we have generated and characterized an immortalized human esophageal myofibroblast line by transfection with a commercially available GFP-hTERT lentivirus. Immortalized human esophageal myofibroblasts demonstrate phenotypic, genotypic and functional similarity to primary cultures of esophageal myofibroblasts we have previously described. We found that immortalized esophageal myofibroblasts retain myofibroblast spindle-shaped morphology at low and high confluence beyond passage 80, and express α-SMA, vimentin, and CD90 myofibroblast markers. Immortalized human esophageal myofibroblasts also express the putative acid receptor TRPV1 and TLR4 and retain the functional capacity to respond to stimuli encountered in GERD with secretion of IL-6. Finally, immortalized human esophageal myofibroblasts also support the stratified growth of squamous esophageal epithelial cells in 3D organotypic cultures. This newly characterized immortalized human esophageal myofibroblast cell line can be used in future cellular signaling and co-culture studies.

## Introduction

Gastro-esophageal reflux disease (GERD) is one of the most common causes of chronic esophageal injury. Mucosal injury stems from refluxed gastric acid, pepsin and bile[1] along with possible shifts in the microbiome [2]. However the cellular and molecular mechanisms governing inflammation and epithelial proliferation in GERD injury are ill-defined. Human myofibroblasts are sub-epithelial stromal cells with a recognized role in the regulation of epithelial proliferation and inflammation via paracrine signaling [3]. They are characterized by expression of cytoskeletal proteins α-SMA and vimentin and cell surface protein CD90 [4]. Stromal cells with a myofibroblast phenotype present in the normal human esophagus are increased in individuals with gastro-esophageal reflux disease (GERD) [5]. Using primary cultures of myofibroblasts established from normal human esophagus in in vitro models of GERD injury [5] we have previously demonstrated that myofibroblasts stimulated with acid and toll-like receptor 4 (TLR4) agonists increase secretion of IL-6. Use of primary cell cultures in biological studies has the advantage of reflecting the in vivo environment. However a relatively short life span secondary to a limited capacity to replicate, and unavoidable heterogeneity amongst primary cultures limits the usefulness of this model in larger scale in vitro cellular signaling studies.

Primary esophageal myofibroblasts grown on plastic typically either senesce or become transformed within 25 passages. The recent advances in our understanding of the potential role for esophageal myofibroblasts in the pathogenesis of GERD injury [5,6] requires a model for studying GERD injury that is not hampered by the limitations of primary cells. Consistent and reproducible studies are particularly critical for signaling studies of understudied cellular populations such as esophageal myofibroblasts.

The major aim of this paper was to generate a human esophageal myofibroblast line with an extended lifespan to facilitate future investigation of the mechanisms by which human esophageal myofibroblasts respond to stimuli encountered in esophageal inflammatory disorders such as GERD (e.g. acid and TLR4 ligands) in the complex environment of the esophageal mucosa. Based on literature review [7,8] we chose to immortalize esophageal myofibroblast primary culture by ectopic expression of human telomerase reverse transcriptase (hTERT). Functional immortalization of normal human esophageal myofibroblasts by telomerase expression does not require the use of viral oncogenes which have ill-defined cellular effects and does not lead to altered patterns of growth such as loss of contact inhibition and acquisition of serum-independent growth. Further GFP expression allows for reproducible identification and sorting of myofibroblasts in multi-cellular studies.

## Methods

### Transfection

Primary cultures of esophageal myofibroblasts [5] were established from de-identified normal human esophageal specimens from donors without a known history of esophageal disorders. Histologic review of resected specimens demonstrated normal esophagus without histopathologic criteria of GERD [5] or Barrett’s esophagus. This protocol was approved by the Institutional Review Board of Keck School of Medicine of University of Southern California and deemed coded specimens/data. Ready to use lentivirus containing human telomerase (hTERT) was purchased from Biogenova (Potomac, Maryland). The viral infection of primary esophageal myofibroblasts [5] established from one normal human esophagus was done according to the manufacture’s protocol. Briefly, 5 x 105 cells were plated into a well of a 6-well plate 24 hours prior to transduction. The Lenti-hTERT-GFP virus containing polybrene (final concentration 8ug/ml) was mixed 1:1 with previously described myofibroblast growth medium [5] and added to the culture after removing old medium. After overnight exposure, the virus was replaced with normal growth medium and cells were allowed to reach confluence. The transfected cells were then sorted by GFP expression on a BD FACSAriaII cell sorter. Cells with strong GFP expression (0.1%) were sorted and replated in culture dishes in previously described myofibroblast media[5]. Briefly, myofibroblast media consists of DMEM with 10% FBS, 10 mg/ml insulin, 10 μg/ml transferrin (Roche, Basel, Switzerland), 10 g/ml gentamicin, and 2 ng/ml EGF (Sigma-Aldrich, St. Louis, MO). GFP-hTERT transfected cells were adherent to plate bottoms and were passaged when confluence reached 80% using trypsin EDTA.

### Cell Culture

Primary esophageal myofibroblasts and GFP-hTERT transfected esophageal myofibroblasts were cultured in myofibroblast media as previously described and incubated in 37°C with 5% CO2. Treatment with acidified media and LPS was performed as previously described [5]. Briefly, cells grown in 12 well plates (5.0 x 10^4^ cells/well) were treated with pH 4.5 serum-free myofibroblast media for 15 minutes followed by replacement with fresh serum-free myofibroblast media and collection of media at 6 hours. Cells were treated with 10 μg/ml LPS for 24 hours and conditioned media was collected. IL-6 ELISA was performed according to manufacturer’s instructions (R&D, Minneapolis, MN).

### Flow cytometry

Flow cytometry was performed to further characterize hTERT transfected cells. Antibodies to CD90 conjugated to APC, CD45 conjugated to PE-Cy7, CD31 conjugated to eFluor450, and CD324 conjugated to PerCP710 were purchased from eBioscience (San Diego, CA). Briefly, single- and multi-color immunostaining was performed according to standard surface FACS staining protocols (eBioscience). Cells were analyzed by flow cytometry using LSRII cytometer (BD Biosciences, San Jose, CA) according to the manufacturer’s protocol. Forward and side scatters thresholds were used to exclude debris and discriminate single live cells. Viability was also assayed using Annexin V and 7-aminoactinomycin D (BD Pharmagen, San Jose, CA) and an additional gate was used to exclude dead cells from the analysis. Flow cytometry data were analyzed with FlowJo software (Tree Star, Ashland, OR). Appropriate isotype controls were used to determine nonspecific antibody staining.

### Immunofluorescence

Immunocytochemistry for α-SMA, vimentin, TLR4 (Abcam, Cambridge, MA) and TRPV1 (Santa Cruz, Dallas, TX) were performed on transfected myofibroblasts grown in chamber slides at several passages as previously described [5]. Cells were examined with fluorescence (TE 300 Nikon Eclipse; Nikon Instruments, Melville, NY) and confocal laser-scanning (LSM 510; Zeiss, Thornwood, NY) microscope. Fluorescent images were acquired using a 40x oil-immersion objectives.

### RNA Isolation and RT-PCR

RNA was harvested from transfected myofibroblasts at several passages using the GeneElute MammalianTotal RNA Mini-prep Kit (Sigma-Aldrich), per manufacturer’s instructions.

cDNA was synthesized and mRNA expression of the following genes was evaluated: α-SMA (forward: CTG TTC CAG CCA TCC TTCAT; reverse: CCG TGA TCT CCT TCT GCA TT), vimentin (forward: TGT CCA AAT CGA TGT GGA TGT TTC; reverse: TTG TAC CAT TCT TCT GCC TCC TG), hTERT (forward: TGGTTTCTGTGTGGTGTCA; reverse: TTGTCGCCTGAGGAGTAGA), TRPV1 (forward: GAC TTC AAG GCT GTC TTC ATC ATC C; reverse: GAC TTC AAG GCT GTC TTC ATC ATC C), TLR4 (forward:CAG GGC TTT TCT GAG TCG TC; reverse TGA GCA GTC GTG CTG GTA TC). Human 18s (forward CCA TGA AGA GGT GAG CGG GGA TTG; reverse ATT AAG TCC CTG CCC TTT GTA CAC) was used as the endogenous control to normalize samples using the ΔΔCT method of relative quantitation, where CT is the threshold cycle. Expression data is presented in log form. Primary esophageal myofibroblasts were used as controls.

### 3D ALI OTC

Human esophageal epithelial cell line EPC2 (a human telomerase reverse transcriptase-immortalized esophageal cell line that was a kind gift from Dr. Anil Rustgi, University of Pennsylvania, Philadelphia, PA) were cultured in a 3D air-liquid interface (ALI) organotyic culture (OTC) system utilizing primary and GFP-hTERT esophageal myofibroblasts in the collagen matrix support layer. Culture conditions were as per published methods [9] with the following modification for the use of human esophageal myofibroblasts. Esophageal myofibroblasts (5x10^5 cells at 6x10^5/ml) mixed with collagen type I (Advanced Biometrics, Delta, BC, Canada) and matrigel (Corning, NY) were cultured for 7 days in myofibroblast medium described above. Myofibroblast-mediated constriction of the collagen matrix was observed. Additional required culture medium was prepared, squamous epithelial cells were seeded, and the air-liquid interface was established according to published methods[9].

### Statistics

All experiments were performed in triplicate and data presented as means ± SE. Data were analyzed using Student’s two-tailed type 2 *t*-test (Excel; Microsoft, Redmond, WA and GraphPad Prism 6, La Jolla, CA).

## Results

**Fig 1:**
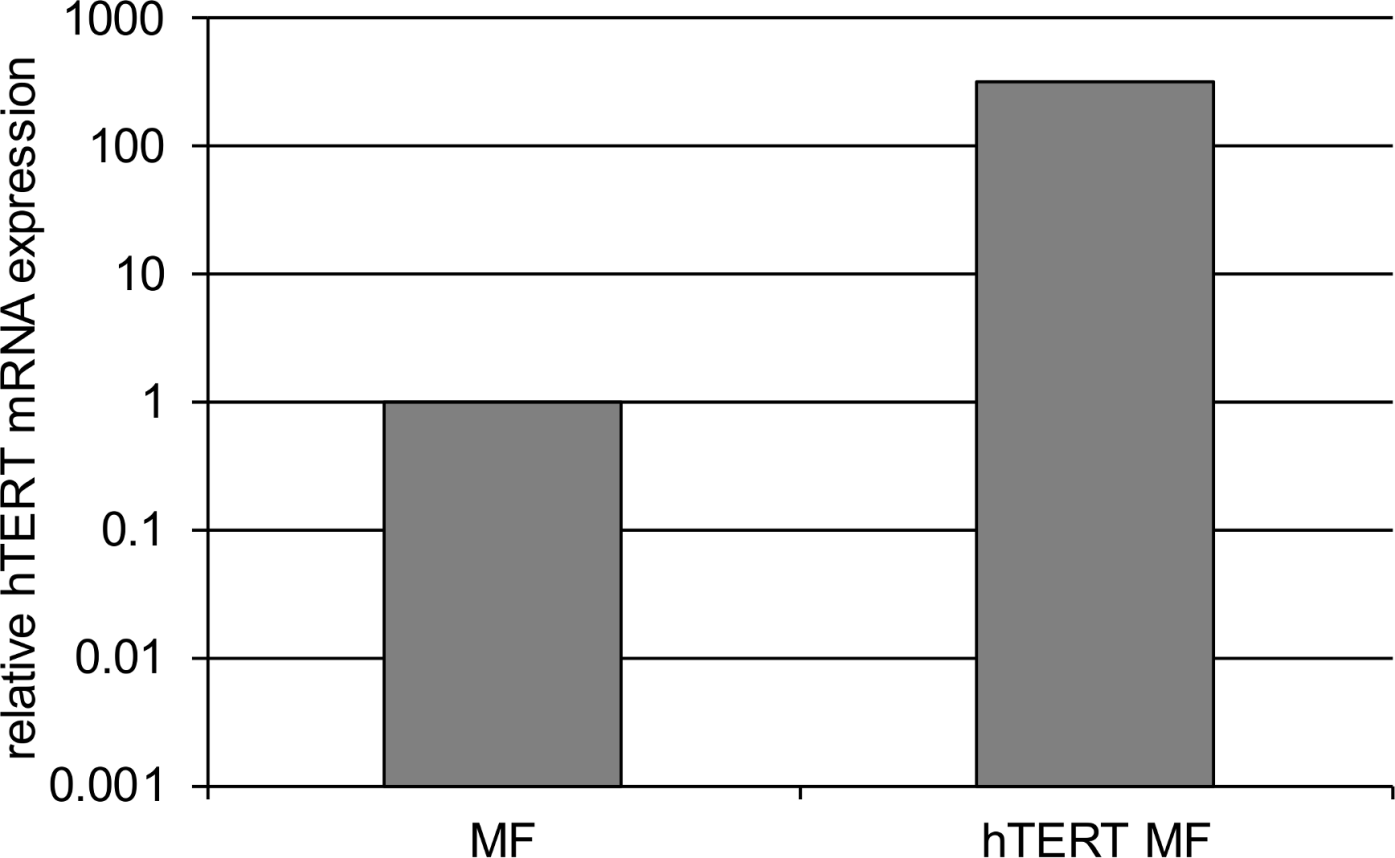
hTERT expression in immortalized esophageal myofibroblasts. Primary esophageal myofibroblasts transfected with human telomerase (hTERT) express elevated hTERT transcript levels as determined by quantitative RT-PCR.

We generated immortal esophageal myofibroblasts by transfecting previously characterized early passage primary esophageal myofibroblasts established from normal human esophagus [5] with a commercially available replication incompetent lentiviral vector encoding *hTERT and eGFP*. hTERT is driven by a CMV promoter and GFP is driven by a separate EF1a promoter. Flow cytometry followed by FACS was performed on transfected cells to sort out GFP expressing cells. Up to 0.1% of cells expressed GFP. GFP expressing cells were cultured and evaluated for hTERT expression by quantitative RT-PCR. Elevated hTERT transcript levels were detected in GFP-hTERT transfected human esophageal myofibroblasts (**Fig 1**).

**Fig 2:**
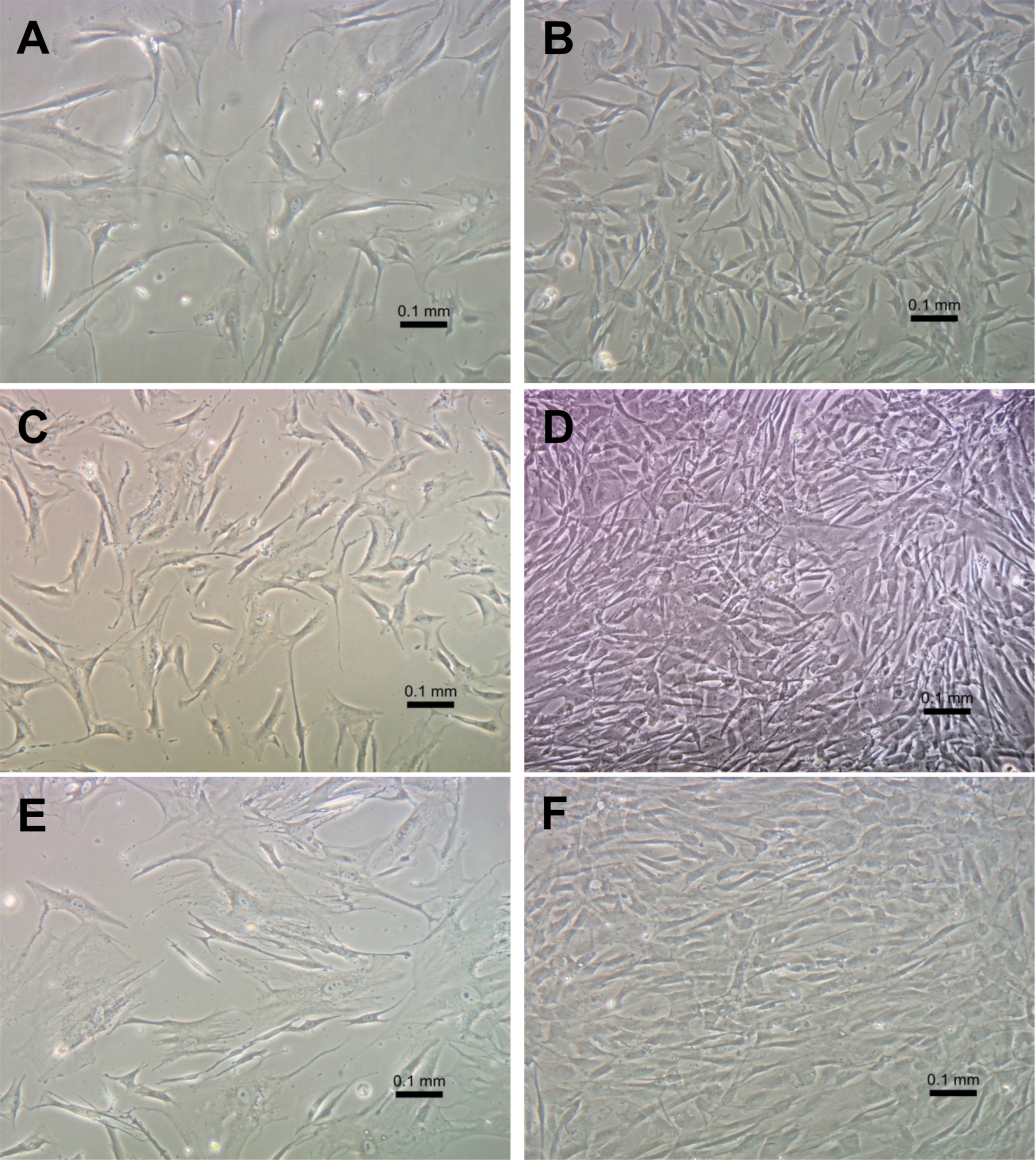
Morphology of primary esophageal myofibroblasts and GFP-hTERT transfected human esophageal myofibroblasts in culture. GFP-hTERT transfected human esophageal myofibroblasts were examined with an inverted microscope and compared to morphology of primary cultures of esophageal myofibroblasts. Representative images of GFP-hTERT transfected myofibroblasts obtained at passage 5 (**A, B**) and passage 89 (**C, D**) at low and high confluence are shown. Representative images of primary human esophageal myofibroblasts at passage 8 in culture at low and high confluence are shown (**E, F**) (magnification 200x). Scale bar represents 100 μm.

We then evaluated the morphology of cultured GFP-hTERT transfected cells by examination with an inverted microscope. Morphology of transfected myofibroblasts was similar to that of primary cultures from which they were established (**Fig 2**). Similar to primary cultures, transfected cells at low passage demonstrated spindle-shaped morphology at low and high confluence. Transfected esophageal myofibroblasts examined up to passage 89 also maintained myofibroblast morphology at low and high confluence and did not senesce, consistent with immortalization. Overall, immortalized esophageal myofibroblasts did not demonstrate identifiable changes in morphology between early and later passages, remained dependent on tissue-culture treated plates and grew in a monolayer. They did not grow in 0.1% serum and required myofibroblast-media used for primary cells for growth. Telomerase expressing human esophageal myofibroblasts remained dependent on solid support for growth.

**Fig 3:**
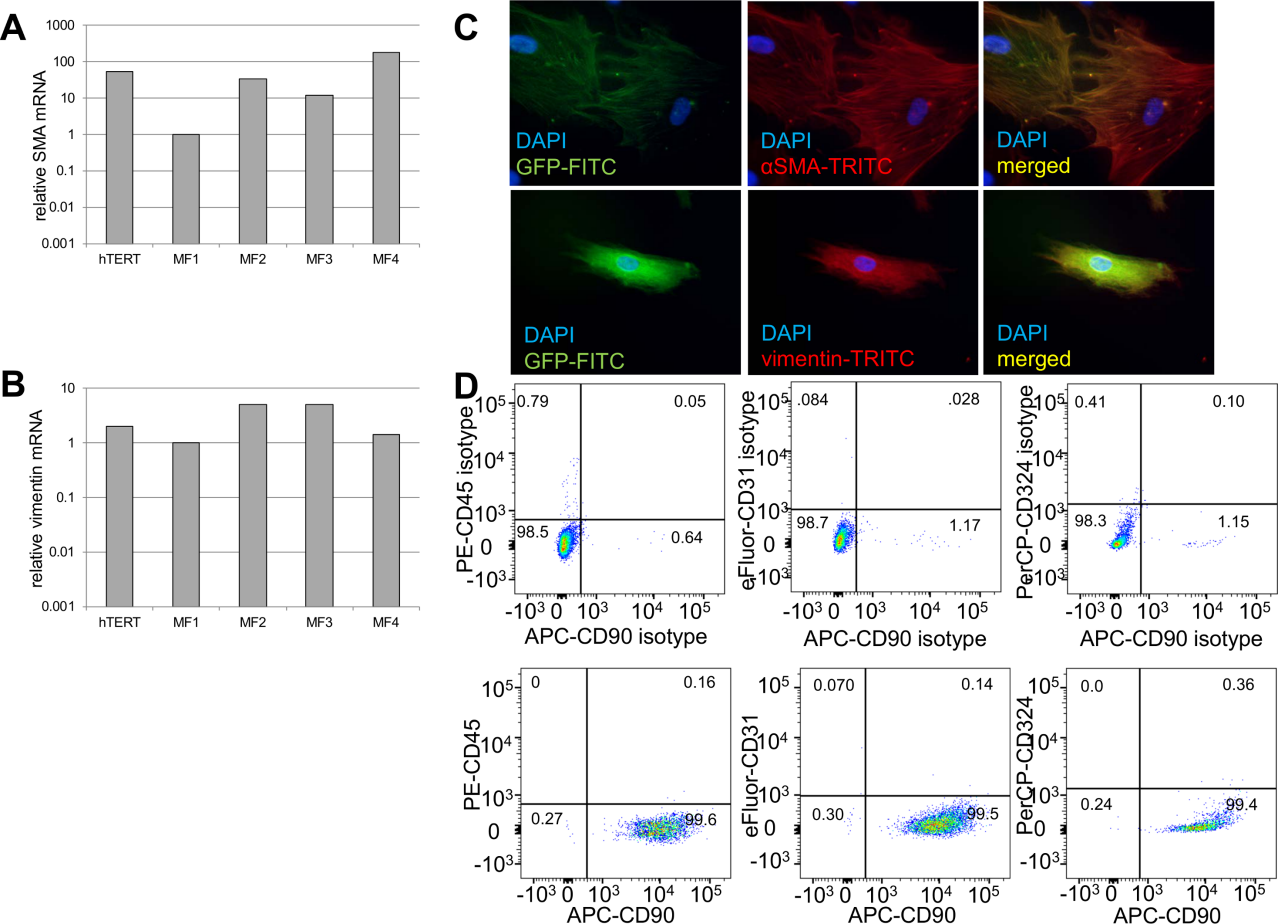
GFP-hTERT transfected esophageal myofibroblasts express myofibroblast markers and lack hematopoietic, endothelial, and epithelial markers. qRT-PCR for myofibroblast markers demonstrates GFP-hTERT esophageal myofibroblasts express α-SMA (**A**) and vimentin (**B**) mRNA. The variability in α-SMA mRNA expression across primary cultures was more profound than that of vimentin mRNA expression. Immunofluorescent staining performed on GFP-hTERT transfected cells grown on chamber slides (**C**) demonstrates co-expression of GFP and α-SMA (top row) and GFP and vimentin (bottom row). Flow cytometry (**D**) for CD90, CD45, CD31, and CD324 (bottom row) with appropriate isotype controls (top row) demonstrates that GFP-hTERT cells express stromal marker CD90 and do not express hematopoietic, endothelial, and epithelial markers cell surface markers.

SMA and vimentin mRNA expression was variable across myofibroblast primary cultures as previously reported and easily detected in hTERT transfected immortalized MFs (**Fig 3A and 3B**). Protein expression of these cytoskeletal proteins was readily detected in immortalized MFs (**Fig 3C**) and was similar to that previously described for primary myofibroblasts [5]. As expected, expression of GFP was readily observed under the immunofluorescence microscope in immortalized cells. Immortalized esophageal myofibroblasts expressed stromal marker CD90 and did not express cytoskeletal, endothelial, or hematopoietic cell surface markers (**Fig 3D**).

**Fig 4:**
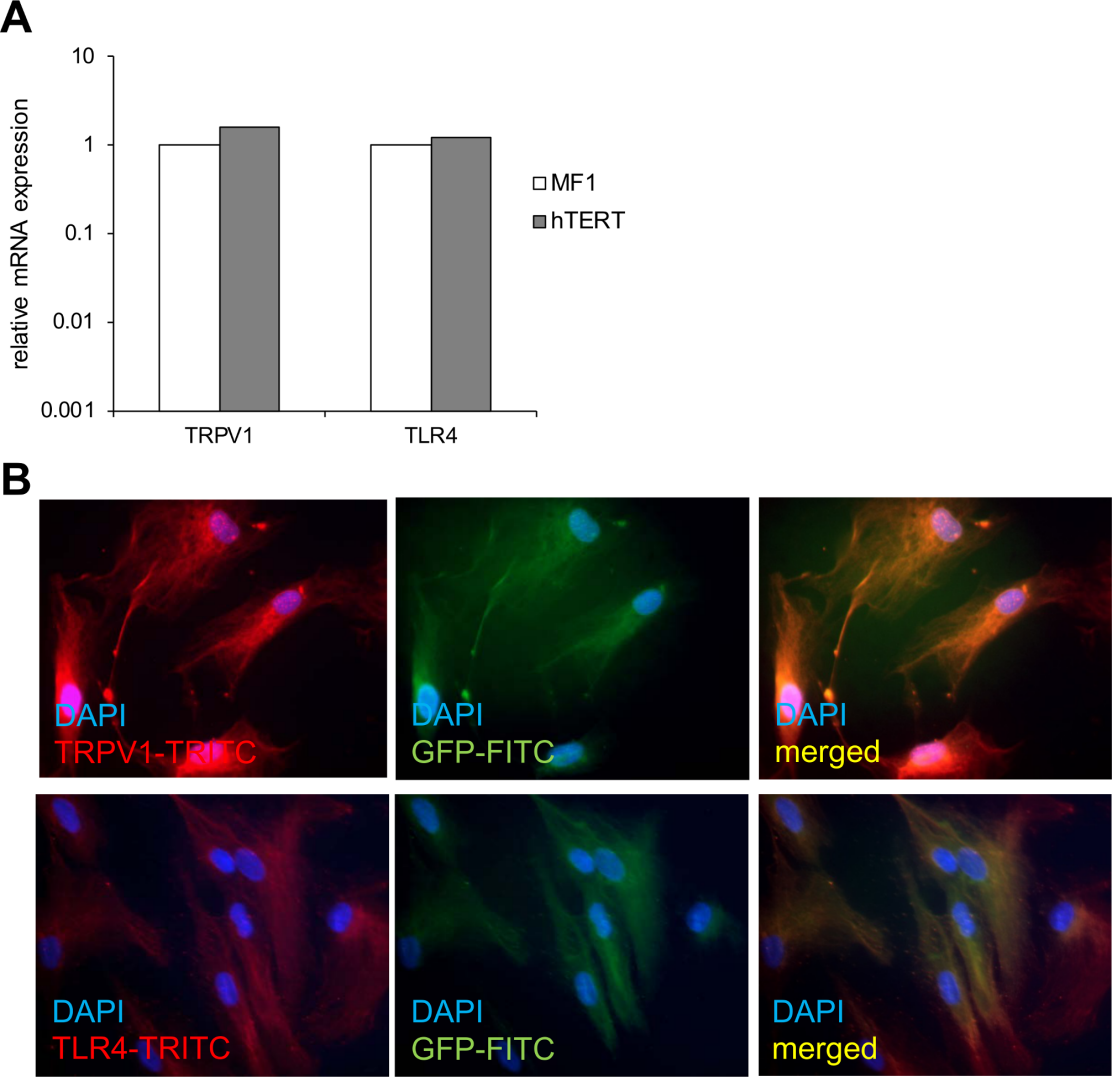
GFP-hTERT transfected cells express TRPV1 and TRL4. qRT-PCR demonstrates similar mRNA expression of TRPV1 and TLR4 in primary esophageal myofibroblasts and GFP-hTERT transfected human esophageal myofibroblasts (**A**). Immunofluorescent staining performed on GFP-hTERT transfected cells demonstrates co-expression of GFP and cytoplasmic staining of TRPV1 (top row) and TLR4 (bottom row) Images examined with a fluorescent microscope. (**B**).

We then evaluated expression of two receptors for injurious luminal and epithelial derived signals encountered in GERD in transfected cells. We have previously shown that primary cultures of esophageal myofibroblasts express transient receptor potential channel vanilloid subfamily 1 (TRPV1) and TLR4. TRPV1 is the putative acid receptor and TLR4 is responsible for signaling in response to gram negative bacteria and DAMPs [5]. TRPV1 and TLR4 mRNA was readily observed in immortalized esophageal myofibroblasts, similar to primary human esophageal myofibroblasts. Immunostaining for TRPV1 and TLR4 protein expression was also performed on immortalized esophageal myofibroblasts. Images were examined with a fluorescent and confocal microscope (data not shown) and showed similar pattern of cytoplasmic staining of these proteins in primary cultures and immortalized cells (**Fig 4**).

**Fig 5:**
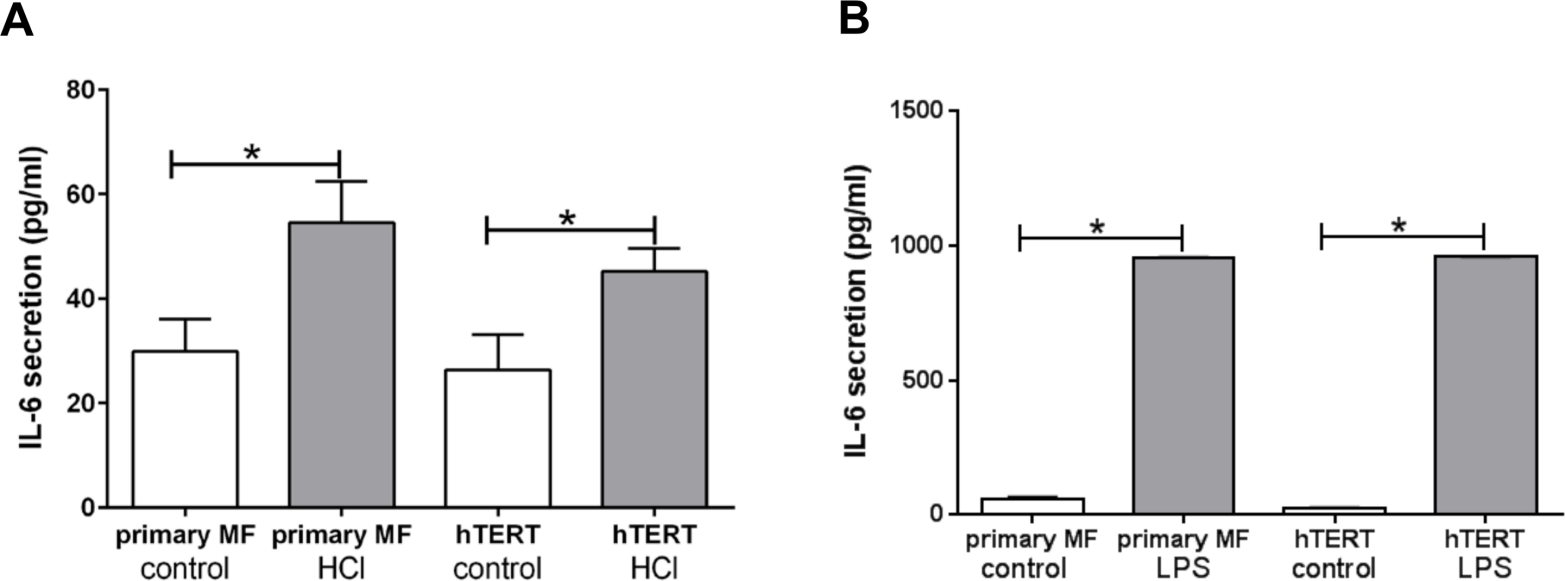
GFP-hTERT transfected cells secrete IL-6 in response to treatment with acidified media and TLR4 ligand LPS. Primary cultures of human esophageal myofibroblasts and GFP-hTERT transfected esophageal myofibroblasts were treated with pH 4.5 acidified serum-free media for 15 minutes followed by culture in serum-free myofibroblast media for 6 hours (**A**) and with LPS 10mg/ml for 24 hours (**B**). Similar to primary myofibroblasts, GFP-hTERT transfected esophageal myofibroblasts increase IL-6 secretion in response to acidified media and LPS. IL-6 was performed on conditioned media. Results represent the means ± SEM of three individual experiments performed in primary cultures and in GFP-hTERT transfected cells. P values were determined by unpaired, 2 tailed T-test. * P < 0.05.

We had previously reported that primary esophageal myofibroblasts increase secretion of IL-6 in response to pH 4.5 acidified media and TLR4 ligand LPS. We therefore evaluated the response of immortalized human esophageal myofibroblasts cells to treatment with pH 4.5 acidifed media and TLR4 ligand lipopolysaccharide. Similar to primary esophageal myofibroblasts, immortalized cells increase IL-6 secretion (26.4 pg/ml vs. 45.2 pg/ml, p < 0.05) in response to acid and LPS (26.2 pg/ml vs. 958.4 pg/ml, p < 0.05) (**Fig 5**). A more profound increase in IL-6 secretion in response to LPS in comparison to treatment with acidified media was observed in immortalized esophageal myofibroblasts. This pattern has been previously reported in primary esophageal myofibroblasts [5].

**Fig 6:**
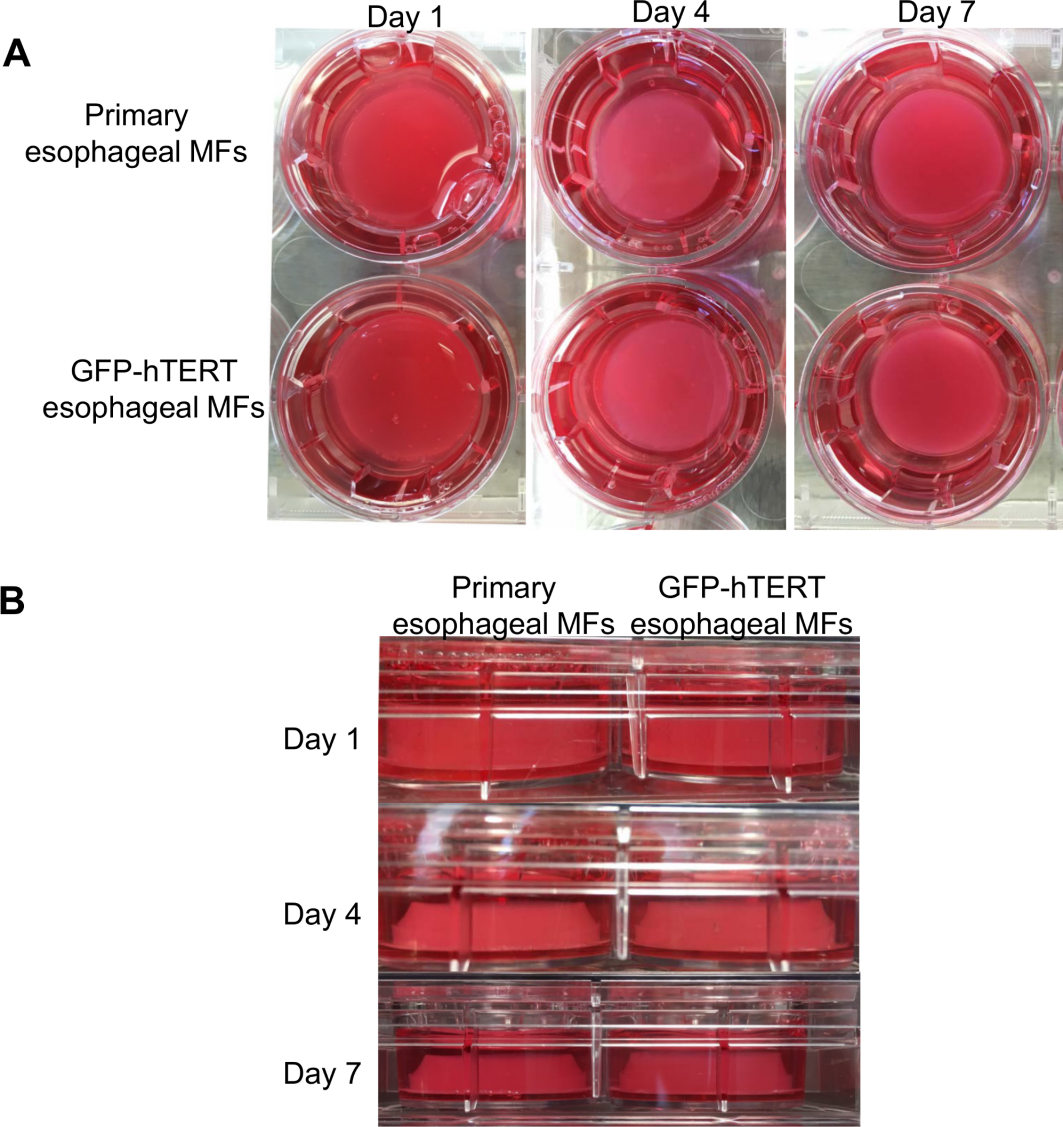
GFP-hTERT transfected esophageal myofibroblasts constriction of the collagen matrix. Primary cultures of esopahgeal myofibroblasts and GFP-hTERT transfected esophageal myofibroblasts were cultured into a collagen matrix as per [9] in 6 well plates. Top (A) and side images (B) of the wells were obtained on days 1, 4, and on day 7 prior to seeding of epithelial cells.

We further investigated the functional similarity between primary and immortalized esophageal myofibroblasts in 3D organotypic culture (OTC) that recapitulates stratified squamous epithelium of the esophagus. Immortalized human esophageal cells cultured in collagen constricted the collagen matrix to a similar degree as primary esophageal myofibroblasts (**Fig 6**).

**Fig 7:**
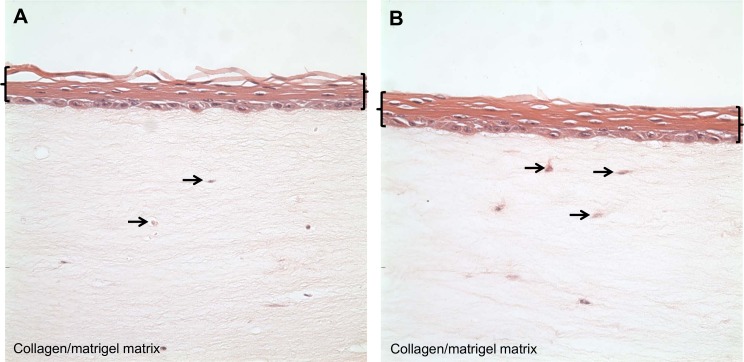
GFP-hTERT transfected esophageal myofibroblasts support the growth of esophageal epithelial cells in 3D OTC. Primary cultures of esophageal myofibroblasts (A) and GFP-hTERT transfected esophageal myofibroblasts (B) were mixed with cellular layer components as per [9] to form a support layer for the growth of EPC-hTERT cells into stratified squamous epithelium. Brackets delineate the stratified squamous epithelium; arrows delineate primary (A) and GFP-hTERT (B) human esophageal myofibroblasts in the collagen/matrigel matrix.

We then showed that immortalized esophageal myofibroblasts support the stratified growth of human esophageal squamous epithelial cells EPC2 (kind gift of Anil Rustgi, University of Pennsylvania) similar to primary esophageal myofibroblasts (**Fig 7**).

## Discussion

Although various approaches to immortalization have been described, ectopic expression of hTERT is a recognized method of overcoming or delaying the senescence that limits the long term use of primary cells [8,10]. Successful immortalization of human fibroblasts without malignant transformation has been previously described [7]. In the work presented here we have generated and characterized an immortalized esophageal myofibroblast line by transfection with a commercially available GFP-hTERT lentivirus. Immortalized human esophageal myofibroblasts demonstrate phenotypic, genotypic and functional similarity to primary cultures of esophageal myofibroblasts we have previously described [5]. GFP-hTERT transfected esophageal myofibroblasts have been cultured beyond 80 passages without evidence of senescence or characteristics of malignant transformation such as lack of dependence on solid support or serum for growth.

We found that immortalized esophageal myofibroblasts retain myofibroblast spindle-shaped morphology at low and high confluence, express α-SMA and vimentin myofibroblast markers, express stromal cell surface protein CD90, and lack expression of hematopoietic, endothelial, and epithelial markers. Immortalized esophageal myofibroblasts also express TRPV1 and TLR4 and retain the functional capacity to respond to stimuli encountered in GERD with secretion of IL-6. Immunostaining demonstrates similar diffuse cytoplasmic expression of these receptors in both primary cultures and immortalized esophageal myofibroblasts. Cell surface expression and the mechanisms by which stimulants gain entrance into the cell are topics of ongoing investigation. Similar to primary cultures, immortalized esophageal myofibroblasts also support the growth of epithelial cells in 3D OTC.

The GFP component of immortalized esophageal myofibroblasts we have generated will allow for effective identification of human esophageal myofibroblasts in multi-cellular and co-culture studies. Immortalization allows for the ability to proceed with cell to cell signaling studies in co-culture or 3D OTC format in a reproducible fashion. Such studies are required to fully delineate the cellular signaling mechanisms mediating complications of GERD related esophageal inflammatory disorders such esophagitis, strictures, intestinal metaplasia and esophageal adenocarcinoma.
